# Historical and Current Ethnographic Use of Tobacco in Brazil: Implications for the Medicinal Use of Nicotine in Parkinson’s Disease

**DOI:** 10.26502/jesph.96120229

**Published:** 2026-08-06

**Authors:** Érica Novaes Soares, Ana Carla dos Santos Costa, Gabriel de Jesus Ferrolho, Rodrigo Portes Ureshino, Silvia Lima Costa, Yousef Tizabi, Victor Diogenes Amaral da Silva

**Affiliations:** 1 Laboratory of Neurochemistry and Cell Biology, Department of Biochemistry and Biophysics, Institute of Health Sciences, Federal University of Bahia, Salvador, Bahia, Brazil.; 2 Laboratory of Neuroscience, Institute of Health Sciences, Federal University of Bahia, Salvador, Bahia, Brazil.; 3 Department of Biological Sciences, Universidade Federal de São Paulo, Diadema, SP, Brazil.; 4 Laboratory of Molecular and Translational Endocrinology, Escola Paulista de Medicina, Universidade Federal de São Paulo, São Paulo, SP, Brazil.; 5 Department of Pharmacology, Howard University College of Medicine, Washington, DC 20059, USA.

**Keywords:** Ethnopharmacology, Tobacco, Nicotine, Nicotinic Receptors, Neuroprotection, Parkinson’s Disease

## Abstract

Global epidemiology data show that disability and death due to Parkinson’s disease (PD) are increasing faster than any other neurological disorder. In 2019, over 8.5 million individuals were diagnosed with PD globally, representing a doubling of cases over the past 25 years. Data of ethnic variation prevalence in US indicate more common occurrence of PD in White people than other ethnicities, although data for “Native Americans,” is not available. Ethnic comparisons in Brazil also indicate more common prevalence of PD in White people, while no such cases have been reported in the indigenous people. In Brazil, this may be related to income concentration and better living conditions for white people, which results in greater longevity, the most important risk factor for Parkinson’s disease. On the other hand, whether this is due to extensive use of tobacco by this ethnic group remains to be seen as numerous epidemiological data indicates an inverse relationship between smoking and PD. Although smoking can result in premature death, nicotine, the addictive component, has not only potent neuroprotective properties but also other therapeutic potentials. Indeed, tobacco has been historically used by indigenous and African-South American people for the treatment of a variety of diseases. Nonetheless, important controlled clinical trials on the effectiveness of nicotine in such diseases are lacking. In this article, we review the use of tobacco by indigenous and Afro-descendants in Brazil as a prelude to the pharmacological use of nicotine in modern medicine. Specifically, we discuss how this knowledge can contribute to a potential novel therapy for PD.

## Introduction

**Figure 1:**
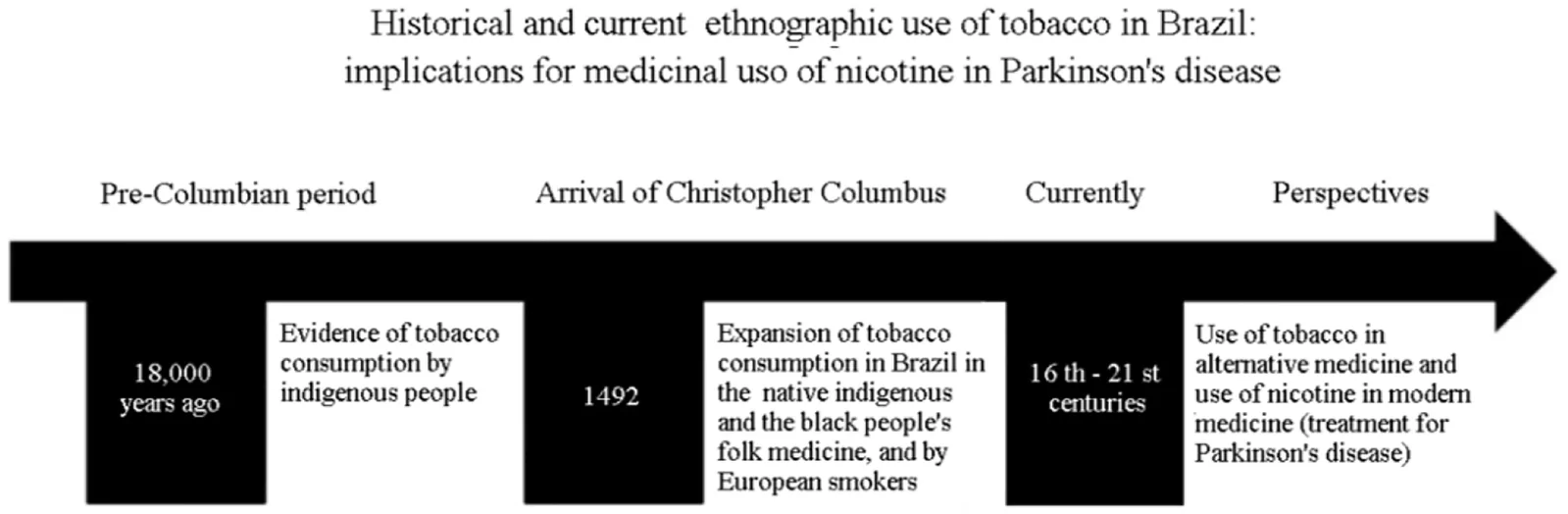
Schematic diagram depicting the historical timeline of tobacco use by indigenous folks in Brazil and implication for the therapeutic use of nicotine in Parkinson’s disease.

*Nicotiana tabacum* L and *Nicotiana rustica* L were found around 18,000 years ago, when humans migrated to the American continent. However, the use of tobacco became more frequent after the arrival of Christopher Columbus in 1492 [1]. Thus, tobacco was already used by indigenous people before the arrival of Europeans on the American continent (Figure 1). It was consumed in different ways, depending on the region, culture and customs of the indigenous ethnicity [1–4]. The purposes of use were also diverse, ranging from divination and shamanistic rituals to treatment of different conditions [2–4]. Tobacco was applied as a tool in Amazonian cultural medicine by local indigenous healers [2–4]. For instance, Peruvian Amazon healers use *Nicotiana rustica* L or Aztec tobacco as their main medicine [3,5], whereas Brazilian endogenous tribes usually consume a mixture containing *Nicotiana tabacum* L and other medicinal herbs such as *Dipteryx odorata* (Aubl.) Willd, *Byrsonima crassifolia* (L.) Kunth, *Platycyamus regnellii* Benth, *Miconia albicans* (Sw.) Steud, *Theobroma cacao* L, *Syagrus coronata* (Mart.) Becc., and *Calycophyllum spruceanum* f. *brasiliensis* K. Schum in their ritualistic activities [6–8]. Interestingly, tobacco also has been used in many Native American rituals including attempts to unify the participants with the spiritual powers and/or honoring the earth and the creator. In addition, tobacco, alone or in association with other plants, is widely used for hygiene and treatment of various health conditions such as dysentery, headache and relief of fatigue by indigenous healers. Curiously, tribes in and adjacent to eastern Bolivia use tobacco to get rid of flies that burrow under the skin of humans [2,4,5].

In addition to the indigenous native people in Brazil, the Afro-Brazilians also frequently use medicinal herbs, including tobacco. The ancestors of this population were brought from different regions and ethnicities of Africa to be employed as slave labor in Brazil. In this immigration process, Africans mixed their own cultural knowledge of the medicinal plants with those of native indigenous peoples, improving the use of Brazilian plants in popular medicine. This knowledge, disseminated between generations, is still present in Afro-Brazilian religions, where the use of plants, among other functions, has medicinal properties, a concept that is directly related to the belief that the disease is not only a biological disorder, but also spiritual. Therefore, plants are believed to hold primordial energy and spiritual properties, and for this reason are used to improve general health and fight against various conditions such as stomach pain, nausea, hypertension, anemia, diabetes, anxiety, depression, and cognitive decline [9,10].

According to the World Health Organization (WHO, 2019), such traditional medicines are defined as “knowledge, skills and practices based on theories, beliefs and indigenous experiences of different cultures, explainable or not, used in the maintenance of health as well as in the prevention, diagnosis and amelioration or treatment of physical and mental illness.” These medicines play an essential role in modern global health in many communities, and the practice of traditional medicine is the most immediate and cost-effective way of health care [4,11]. Therefore, preservation of knowledge on the use of medicinal plants by Amerindian (indigenous peoples of the Americas) and Afro-descendant people can be useful in the prospection of new drugs. However, the search for tobacco-based treatments has not been sufficiently addressed by the scientific community. Thus, following a brief historical review of the human use of tobacco, particularly in folk medicine, we focus on the potential use of nicotine, the primary compound in tobacco, in modern medicine.

### Historical use of tobacco in folk medicine

The historical reports on the use of tobacco by indigenous people in Brazil mention its use as Rapé, which is a ceremonial snuff that is blown into the nostrils using a special bamboo or wooden pipe (Table 1) [2,3,5]. It should be noted that the term “chewing tobacco” does not accurately define the action taken by the South American Indians towards this practice because instead of chewing, they hold the tobacco in their cheeks for a long time while the juice runs down their throat. So, a more appropriate term in this case would be “tobacco sucking” [2]. Tobacco consumption in this modality occurs in the Lesser Antilles, Venezuela, Guyana, Colombia, the Upper Amazon and in eastern Brazil [1–4]. Akawaio people from Guyana have the habit of holding a mass of green tobacco under their lips. The preparation of chewing tobacco involves a mixture with salt or wild honey [1].

The use of tobacco by indigenous tribes from South America has been associated with acute intoxication that includes hallucinations, visions, dreams and communications with “spirits.” The main methods of administration in these rituals are swallowing the smoke, inhaling, smoking intensely fast on a continuous basis, and drinking “tobacco water”, a craft often performed by shamans. The Pataxó indigenous people from Southern Bahia- Brazil consume tobacco as Rapé in a ritual called Awê, in which all tribal members celebrate by painting their bodies, singing and dancing and eating their typical foods [7]. Another frequent method of tobacco use by shamans is to blow tobacco smoke on people, especially on the sick people [2,4] a powder made from dried tobacco leaves (Nicotiana spp) [6]. Rapé is sometimes mixed with plant ashes and tree barks such as cacaueiro (*Theobroma cacao* L), embaúba (*Cecropia pachystachya Trécul*), jatobá (*Hymenaea courbaril* var. *altissima* Ducke-YTLee & Langenh), and amescla (*Protium heptaphyllum* Marchand) and is usually administered in both nostrils, one at a time [7,8,12]. After self-application using the curipe (or kuxipa) instrument, or applied by another person using the tepi instrument, the individual must suspend nasal inspiration for a moment and keep breathing through the mouth. The Rapé residue should not be swallowed but spat out later [7]. The use of Rapé during the Colonial period in Brazil is supported by artifacts found along the Amazon River. Meticulously carved and polished lithic pieces decorated with zoomorphic figures were used to administer Rapé [13]. Production and use of Rapé differ according to the ethnicity and culture of each community. For example, different community may utilize different instruments containing shells, clay, rocks, wood, bone, or feathers. A common feature in production of Rapé is that initially trays are used to dehydrate and roast the red-hot tobacco leaves and then pestles are used to grind these leaves as well as other seeds, bark and roots that are added to the preparation. Afterwards, the powder is stored in mortars and applied using hollow tubes made from bird bones, rolled leaves, pieces of bamboo or monkey ulna bones, all carefully made and adorned, reflecting the artistic expression of each culture [7,12–14].

### Interaction of two cultures

The term Afro-descendant consists of the union of the two words “afro” which refers to Africans and “descendant” which goes back many generations and signifies “of African origin”. African-born people or Afro-descendants have been living in Brazil since the beginning of colonization in the 16th century. They arrived during a historical and sociocultural process known as the African Diaspora for slave labor. In the process of adaptation in the new territory, these people became familiar with local plants and adapted their previous knowledge of medicinal and “magical” properties to the reality of the new continent [15]. In this process, knowledge on health and the traditional religious beliefs of African people was mixed with those of indigenous people [15,16].

In Afro-Brazilian medicine, health is understood in a comprehensive way consisting not only of biological and organic but also of spiritual components [15,17]. It is believed that the disease actually “arises first in the spiritual/energetic body and then manifests itself in the physical body” and that tobacco acts at the spiritual/energetic level [18]. In this scenario, tobacco alone is used topically or by oral ingestion [18]. The effect of tobacco, however, depends on the specific plant material, its preparation and the added ingredients [19].

### Nicotine, the main component of tobacco

Nicotine is one of more than 4,700 compounds in tobacco smoke. However, it is the only known component with addictive properties and sole reason for smoking [20]. Tobacco plants contain the highest levels of nicotine, but other members of Solanaceae, which include tomatoes (*Solanum lycopersicum* L) and eggplants (*Solanum melongena* L), also produce low levels of nicotine. Nicotine production is not confined to nightshade plants as other species such as cauliflower (*Brassica oleracea* L) and papaya (*Carica papaya* L) that do not belong to this category also contain low levels of nicotine. The amount of nicotine extracted varies with the growth stage of the plant, the plant part, and the method of extraction. For example, the tobacco plant synthesizes nicotine in its roots and then stores it in its leaves, where it constitutes 0.3% to 0.5% of the tobacco plant’s dry weight [20,21]. It is believed that plants produce nicotine as a defense mechanism against predators. Indeed, nicotine at higher concentrations is a potent neurotoxin that was used extensively as a pesticide in agriculture [22]. Although such use of nicotine is now prohibited in many countries, nicotine intoxication is still a problem in tobacco farmers, young children and in adults due to accidental or suicidal ingestions of nicotine products [22]. The most serious toxicity caused by nicotine is the paralysis of the muscles, particularly those controlling breathing, which can result in respiratory as well as cardiovascular failure and death. However, other organs including the brain can also be affected.

### Mechanism of action of nicotine

The action of nicotine is mediated via nicotinic cholinergic receptors (nAChRs), which act by directly regulating the opening of a cation channel allowing for the influx of sodium (Na^+^) and calcium (Ca^2^^+^) [23–25]. The nAChRs are pentamers composed of different combinations of polypeptide chains, primarily as alpha (α) and beta (β) subunits. There are also delta, gamma, and epsilon, that are defined by their developmental stage and are explicitly expressed in the neuromuscular junction. To date, 17 such receptors encoded by a multigene family have been identified [23,25]. These receptors are not only present in the neuromuscular junctions, but also in autonomic ganglia as well as in the central nervous system (CNS). The subunit structures of these receptors, however, are different from each other in different areas. For example, as mentioned above, only the neuromuscular receptors express the delta, gamma or epsilon subunits, whereas the autonomic ganglia and CNS nAChRs are composed of alpha and beta subunits in unique combinations. The most predominant and most extensively studied receptor subtype in the brain, with a high affinity for nicotine or acetylcholine, commonly referred to as “high-affinity binding site,” is formed from α4 and β2 subunits. The other major class, formed from homomeric α7 subunits, is commonly referred to as “low-affinity binding site,” due to its low affinity for acetylcholine [23]. Ganglionic receptors are predominantly composed of α3 and β4. Nicotinic receptors were one of the first receptors identified, hence a vast literature on history and scientific evolution of these receptors are available [23,25,26].

### Role of nicotinic receptors

It is now evident that nicotinic receptors not only play important roles in neuronal functions but may also offer therapeutic targets for various neurodegenerative and neuropsychiatric disorders including Parkinson’s disease (PD) [27–29], mild cognitive impairment or Alzheimer’s disease [26,30–33], multiple sclerosis [34], depression [28,35,36], ischemia [33], schizophrenia [37], pain [38] as well as energy balance [39]. Moreover, these receptors are abundantly expressed in a variety of immune cells including B cells, T cells, macrophages and microglia and are believed to contribute to anti-inflammatory effects of nicotine [40,41]. Indeed, nicotine has been shown to inhibit pro-inflammatory cytokines such as TNF- α, IL-1, and IL-6, without affecting the anti-inflammatory cytokines such as IL-10 [42,43]. This effect of nicotine, in addition to its interaction with angiotensin-converting enzyme 2 (ACE2) via nicotinic receptors has led to the suggestion of a potential role of selective nicotinic receptors in interfering with SARS-Cov2 virus entry and hence improving COVID-19 condition [44]. Therefore, modulation of the nicotinic receptors by appropriate concentrations of nicotine may be exploited for therapeutic purposes.

### Parkinson’s disease

Parkinson’s disease is the second most common progressive neurodegenerative disorder with global epidemiological data showing over 8.5 million individuals afflicted with this devastating motor as well as non-motor symptoms [45]. Ethnic comparison of PD prevalence in US shows more incidence in White compared to other people, although data for “Native Americans” is not available, likely due to small numbers of subjects or race ambiguity [46]. Ethnic comparisons of PD prevalence in Brazil also show more incidence in White people followed by Black people, though curiously, no cases in the indigenous population have been reported [47].

This discrepancy, especially in the Indigenous people, might be due to a lower life expectancy in this population underscored by poorer health and social outcomes [48]. On the other hand, potential contribution of medicinal plants such as extensive use of tobacco in this scenario cannot be ruled out. Thus, further epidemiological data and their careful evaluation is needed. This is because approximately 15% of PD patients have a hereditary form and 5–10% have a monogenic mendelian form with a minimum of 23 loci and 19 causative genes [49,50]. Moreover, contribution of environmental or endogenous toxins as other causative factors requires further investigation.

The main pathological alterations in PD are the accumulation of Lewy bodies, composed mainly of alpha-synuclein, and the loss of dopaminergic neurons in the substantia nigra pars compacta (SNpc) that leads to striatal dopamine (DA) deficiency in basal ganglia [29,51–53]. This dopaminergic loss results in motor deficits characterized by akinesia, rigidity, resting tremor and postural instability as well as non-motor symptoms that might also involve other neurotransmitter systems [27,54,55]. The non-motor symptoms may include cognitive deficits (e.g., mild to severe memory impairment), emotional changes (e.g., depression, apathy, and anxiety), sleep perturbations (e.g., insomnia/hypersomnia), autonomic dysfunction (e.g., bladder disturbances, orthostatic hypotension, sweating), sensory symptoms (e.g., pain, visual and olfactory deficits) and gastrointestinal symptoms (e.g., constipation, nausea) [54,56,57]. The most common treatment for PD is focused on dopamine replacement (e.g., levodopa = L-Dopa). However, not only the efficacy of this drug is invariably reduced in a few years, but long-term treatment may also cause severe dyskinesia or involuntary movements [27,58,59]. Hence, extensive effort in finding novel therapies is ongoing.

### Nicotine for PD

Multiple studies indicate that the normal function of the basal ganglia is dependent on the equilibrium between the midbrain dopaminergic and striatal cholinergic systems. Thus, acetylcholine (ACh) can regulate striatal DA release via interaction with nicotinic receptors [27,29]. Moreover, in various animal models of PD (e.g., 6-OHDA lesioned rodents) the impairment in DA release appears to be exacerbated by a loss of nAChRs, suggesting that nicotinic agonists may ameliorate the dopaminergic imbalance and may thus be useful therapeutic targets for PD. Indeed, several *in-vitro* and *in-vivo* studies in rodents and primates including genetically modified mice, have shown protective effects of nicotine against neuronal damage induced by 6-OHDA, MPTP, rotenone, paraquat, methamphetamine, glutamate and β-amyloid [60]. Nicotine may also protect against salsolinol-induced toxicity in SH-SY5Y cells [61]. Salsolinol is an endogenous product of aldehyde and dopamine condensation with selective toxicity to dopaminergic neurons, which are represented by SH-SY5Y cells [61]. These cells, derived from human neuroblastoma are commonly used as a cellular model to investigate novel treatments for PD [62]. Further studies using selective nicotinic receptor subtype antagonists indicated involvement of both alpha4-beta2 and alpha7 nicotinic receptors in the protective effects of nicotine [61]. Similarly, the damage inflicted by aminochrome, a neurotoxic molecule derived from dopamine oxidation in substantia nigra derived RCSN-3 cells, or in astrocytes could also be protected by nicotine pretreatment [63,64]. Protective effects of nicotine against the toxicity induced by manganese and iron in SH-SY5Y cells with implications for PD have also been reported [65]. Additionally, it was shown that nicotine protects PC12 neural cells against 1-methyl-4-phenylpyridinium ion (MPP+) toxicity via activation of alpha7nAChR/PI3K/Trx-1 and suppression of endoplasmic reticulum (ER) stress [66]. In vivo protective effects of nicotine against MPTP-induced dopaminergic dysfunction and degeneration in mice and monkeys have been extensively investigated [67–73]. Curiously, not only neurons but astrocytes [64,74–77] and microglia [78,79], are also affected by nicotine. It was demonstrated that nicotine induces morphological and functional changes in mice astrocytes via nicotinic receptor activity in the hippocampus and substantia nigra [80]. It has been proposed that targeting α7-nAChRs expressed in glial cells specifically may be a novel therapeutic strategy for PD treatment [80,81]. Since neuroinflammation plays a critical role in PD pathology, nicotine suppression of this process could be a major contributor to its potential utility in PD and perhaps other neurodegenerative diseases as well. Given that there is a relatively high comorbid existence between depression and PD, and that nicotinic intervention may also be beneficial in depression, nicotine and/or nicotinic agonists or modulators may have the added advantage of addressing both issues at the same time [28].

Finally, the anti-fibrillogenic and fibril-destabilizing activity of nicotine as well as its ability to promote the clearance of alpha-synuclein, and hence preventing pathological accumulation of this presynaptic protein, may be of critical importance in its inhibition of Lewy bodies [82–87]. As alluded to earlier, accumulation of Lewy bodies, composed primarily of alpha-synuclein, is a hallmark of PD pathology. Indeed, it is believed that synucleinopathy not only contributes to the movement disorders but also to cognitive and social impairment associated with PD [88]. In further support of the contention that targeting α7-nAChRs may be a novel therapeutic strategy for PD treatment [81,80], it was reported that nicotine’s prevention of synucleinopathies may be mainly due to its interaction with α7-nAChRs and inhibition of apoptotic cell death [86].

### Mode of administration: A potential critical factor in nicotine pharmacotherapy

**Figure 2.**
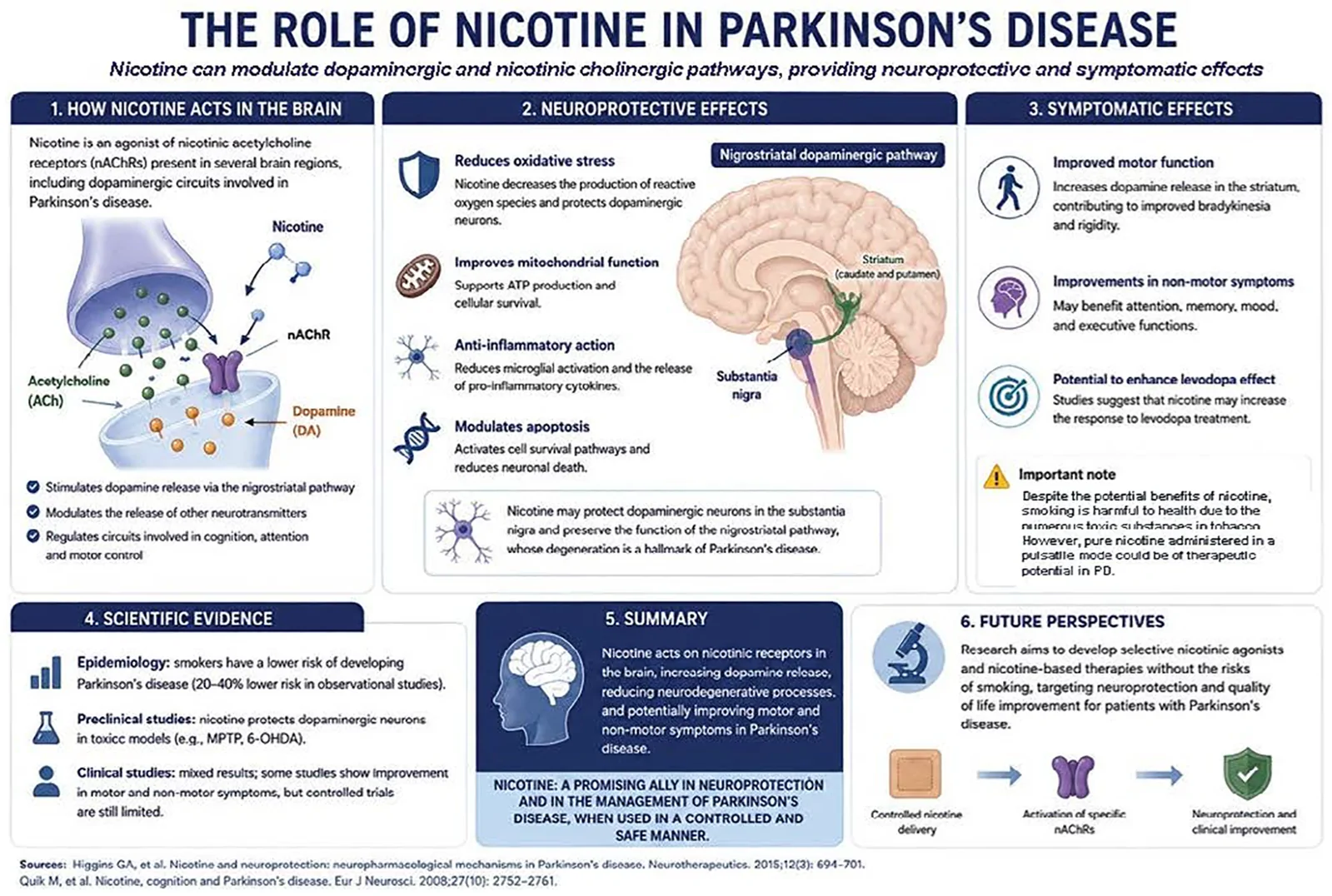
The figure suggests that nicotine by interacting with its receptors in critical brain regions can provide neuroprotection as well as symptomatic relief in Parkinson’s disease (PD). Nicotine in blue, acetylcholine receptors in purple, acetylcholine in green and dopamine in orange (1), neuroprotective effects on substance nigra (2), sympathetic effects (3).

The well-known inverse relationship between smoking and PD and the documented neuroprotective effects of nicotine (discussed above) prompted several clinical trials with nicotine patch in PD [89]. However, except for a single report showing a very modest improvement, no apparent benefit was noted with such mode of nicotine administration [89]. This failure may be due to distinct pharmacokinetic as well as pharmacodynamic differences in nicotine’s mode of administration. Thus, it is likely that the steady release of nicotine delivered from the applied patch can result in prolonged nicotinic receptor desensitization and hence its ineffectiveness in ameliorating PD symptoms [89–91]. Nicotine patch, by maintaining a steady plasma concentration of nicotine, may be an effective intervention for smoking cessation as desensitization of the central nicotinic receptors in critical brain reward circuitry may help ameliorate the withdrawal effects of nicotine. However, such mode of intervention is unlikely to be effective in ameliorating any of PD symptoms. This contention is underscored by the fact that smokers become dependent on nicotine due to stimulation of their central nicotinic receptors as they take a break in between each cigarette as well as between each puff, albeit a very brief break. Hence, it is proposed that a pulsatile stimulation of nicotinic receptors, like that experienced by smokers, is necessary to provide the benefits of nicotine therapy in neurodegenerative diseases in general, and in PD, in particular [60,89]. It is of utmost importance to emphasize that what is being proposed here is delivery of pure nicotine, or a nicotinic agonist/modulator, in a pulsatile mode by routes such as nasal insufflation or inhalation. As mentioned above, tobacco may introduce thousands of chemicals in the body with significant harm. However, nicotine itself as detailed above may not only be effective in ameliorating PD symptoms and even those of L-dopa-induced dyskinesia [92] but may retard the progression of the disease due to its neuroprotective properties (Figure 2).

Still, the addictive properties of nicotine might pose a concern in its therapeutic effects. However, since nicotinic therapy in PD would likely be a prolonged if not life-long intervention, this concern is overshadowed by the risk/benefit consideration which favors significant therapeutic benefit. In addition, efforts in developing selective nicotinic receptor subtype agonists or nicotinic receptor modulators that would be of similar or better potency than nicotine but without its addictive properties could further alleviate this concern [93–95].

## Conclusion

In summary, based on historical and cultural use of tobacco in Brazil and its continuation in modern societies as well as extensive preclinical and limited clinical studies, a pulsatile mode of nicotine administration in PD is suggested.

Table 1 depicts different indigenous Brazilian tribes that use tobacco. This data was compiled according to various studies found in the literature.

## Figures and Tables

**Table 1:** Method of tobacco consumption used by indigenous people in Brazil.

Method of Tobacco Use	Indigenous Tribe	Reference
Chewing	Akawaio; Bora, Caduveo, Carapana, Cayapo, Cocama, Guarani, Kayova, Mashco, Mbayá, Nonuya, Omagua, Patamona, Potiguara, Shavante, Taurepan, Wapishana, Warao, and Yanoama.	[1,2]
Drinking	Akawaio, Aparai, Atorai, Bororo, Campa, Cashinaua, Cocama, Macushi, Mashco, Oayana, Patamona, Taruma, Taurepan, Tupinamba, Wapishana, and Warao.	[2]
Licking	Bora, Campa, Miraña, Muinane, Siona, and Witoto.	[2]
Snuffing (pulsatile inhaling Rapé)	Jamamadi Paumari, Apurinã, Suruwahá, Aicana, Amahuaca, Bora, Cachuena (Kashuiena, +, Campa, Mashco, Miraña, Pano,Paumari, Kahuyana, Campa, Canamari, Carijona, Cashinaua, Cubeo, Guarani, Guararegaja, Kepikiriwát, Maipure, Tucano, Tupari, Waiwai, Witoto, and Yanoama.	[2,7]
Smoking (pulsatile)	Akawaio, Amahuaca, Amanayé, Anauqua (Kuikuru), Aparai, Apiacá, Apinayés (Apinajés), Aramagoto, Arara, Asurini, Atorai, Bacairi, Baniwa, Baré, Bora, Bororo, Botocudo, Caduveo, Caingang, Camacan, Camayura,Campa, Caraja, Carapana, Carib: Guyana, Cashinaua, Cayapo, Chamacoco, Chipaya, Chiquito, Cocama, Cubeo, Cuicuru (Anauqua), Custenau, Desana, Genaken, Guahahara, Guana, Guaraní, Guato, Ingarune, Kalapalo, Kanitana, Kashuiena (Cachuena?),Kaskihá, Katapolítani, Lengua, Macamccra, Macushi, Maipure, Manaje, Manao, Maué, Mayongkong, Mayoruna, Mbayá, Mehinaku, Mojo,Muinane, Mundurucu, Mura, Nambicuara, Omagua, Oyampi, Pano, Parintintin,Patamona, Pira-tapuya, Puri-Coroado, Shavante, Sherente, Siona, Siracua, Siriono (Neozé), Tapirapé, Tapoya, Tapuya, Tarairiu, Tariana, Taruma, Taurepan, Tembé, Tereno, Timbira, Tirio (Trio), Trumai,Tucano, Tupari, Tupinamba, Tuyuka, Uaikana,Urubu, Vilela, Waiwai, Wapishana, Warao, Warekena, Waurá, Wayana (Rucuyen), Witoto, Yawalapiti, Yecuana, and Yuruna.	[2]

## Abbreviations

6-OHDA: 6-hydroxydopamine or oxidopamine
ACE2: angiotensin-converting enzyme 2
Ca^2^+: calcium
CNS: central nervous system
COVID-19: acute respiratory infection caused by the SARS-CoV-2 coronavirus
DA: dopamine
IL-1: Interleukin 1
IL-10: Interleukin 10
IL-6: Interleukin 6
MPP+: 1-methyl-4-phenylpyridinium ion
Na+: sodium
nAChRs: nicotinic cholinergic receptors
PD: Parkinson’s disease
RCSN-3: cells substantia nigra derived
SARS-Cov2: Virus
SNpc: substantia nigra pars compacta
TNF- α: Tumor Necrosis Factor Alpha
α: alpha
β: beta
